# Characterization of the C-terminal tail of the Arc protein

**DOI:** 10.1371/journal.pone.0239870

**Published:** 2020-09-29

**Authors:** Melissa Boldridge, Jody Shimabukuro, Keith Nakamatsu, Christian Won, Chad Jansen, Helen Turner, Lei Wang

**Affiliations:** 1 Department of Natural Sciences, Hawaii Pacific University, Honolulu, Hawaii, United States of America; 2 Department of Natural Sciences, Windward Community College, Kaneohe, Hawaii, United States of America; 3 Laboratory of Immunology and Signal Transduction, School of Natural Sciences and Mathematics, Chaminade University, Honolulu, Hawaii, United States of America; Rijksuniversiteit Groningen, NETHERLANDS

## Abstract

The activity-regulated cytoskeleton-associate protein Arc (or Arg3.1) is specifically linked to memory formation and a number of cognitive disorders, including Alzheimer’s disease and schizophrenia. Since the discovery of Arc in 1995, extensive research has been conducted on the protein to identify its function and mechanisms of action, with solving the structure of Arc as a major goal. However, the Arc protein tends to self-oligomerize *in vitro*, and is difficult to crystallize. These properties have hindered efforts to obtain the structure of the full-length, whole protein Arc. As an alternative approach, we and others, have sought to solve the structures of various subdomain proteins of Arc, including the N-lobe, C-lobe, and capsid domain (N-lobe + C-lobe). In this study, we characterized the C-terminal tail of Arc using integrated bioinformatic and structural biology techniques. We compared the sequences of Arc proteins in different mammal species and found that the amino-acid composition in the C-terminal tail region has a significantly higher degree of variation rate than the rest of the protein. Structural prediction programs suggested that the C-terminal tail is structurally disordered. Chemical shift analysis based on solution NMR spectra confirmed that the C-terminal tail has a random coil (disordered) structure, and the tail starts from the residue D357. Furthermore, the NMR spectra showed that the C-terminal tail has minimum (if any) interaction with its neighboring capsid domain in Arc. This study fills gaps in our specific understanding of the structural nature and functional contributions of the Arc C-terminus.

## Introduction

Long-term memory is believed to be encoded in long-term synaptic plasticity, which requires rapid synthesis of new mRNAs and proteins. While examining newly synthesized biomolecules during memory formation, two research groups independently discovered the 3.1 kb mRNA that encodes the activity-regulated cytoskeleton-associate protein Arc (or Arg3.1) [1, 2]. The Arc gene belongs to the immediate-early gene family, which mediates prompt transcriptional responses to neuronal activities [1]. Arc mRNA and protein are mostly expressed in the hippocampal and parietal cortex neurons that are responsible for learning and memory formation [3]. In these neurons, Arc mRNA selectively accumulates at regions that contain recently activated synapses, and migrates away from inactive synapses [4]. Similar to its mRNA, Arc protein tends to accumulate at activated synapses, and is especially enriched in the postsynaptic density [5]. The distinctive pattern of Arc's selective accumulation at activated synapses suggests its connection with memory formation. Indeed, during behavioral learning, Arc was quickly and robustly induced in rat brains, and was used to mark neuronal networks involved in learning [6, 7]. Arc-knockout mice showed substantial deficits in long-lasting memories for implicit and explicit learning tasks, but their short-term task acquisition memories were not affected [8]. Moreover, antisense inhibition of Arc expression in rat hippocampi resulted in impaired long-term synaptic potentiation and long-term memory consolidation [9]. Extensive research has been conducted on the Arc protein to identify its functional mechanism at the molecular level. Studies have shown that Arc can interact with endocytic machinery, especially with dynamin and endophilin, to regulate neuronal trafficking of AMPA receptors [10]; Arc can interact with the γ-secretase subunit, presenilin, to regulate β-amyloid generation [11]; Arc binds to CaMKII and localizes at silenced synapses [12];Triad3A can bind and ubiquitinate Arc and cause its rapid degradation [13]; the nuclear transportation of Arc is regulated by its nuclear localization, retention, and exportation domains [14]; Arc bears a capsid domain and can form retroviral capsid-like structures to transfer mRNA [15–17].

Solving the structure of Arc could help provide insight into its functions at the residue-specific level. The Arc protein in most mammal species has around 396 amino-acid residues; it tends to self-oligomerize in solution *in vitro* [18], and is difficult to crystallize. These properties have hindered the effort to obtain the structure of the full-length Arc as a whole protein using the traditional x-ray crystallography and solution NMR methods. As an alternative method to obtain Arc’s structure, scientists have produced various subdomain proteins of Arc and solved their structures, respectively. For example, Zhang et al. produced the N-lobe subdomain of rat Arc (Arc^207-277^) and solved the crystal structure of the N-lobe complexed with the peptides derived from TARPγ2 or CaMKIIα. They found that the N-lobe folds into a four-helix bundle, and the peptide binds to a groove of the helix bundle that is formed by its N-terminal β-strand, the loop connecting α-2 and α-3 helices, and the N-terminus of the α-3 helix. They also produced the C-lobe subdomain of Arc (Arc^278-370^) and solved its crystal structure, which folds into a five-helix bundle. They further proposed that the two-lobe arrangement of Arc resembles the capsid domain of the retrovirus-like gag protein [15]. Later, Hallin et al. produced a full-length rat Arc protein by extracting the protein from inclusion bodies, and identified the relative positions of the subdomains of Arc using the small-angle X-ray scattering (SAXS) method. They found that Arc’s coiled-coil subdomain (Arc^26-130^) lies above its bi-lobe subdomain (Arc^210-361^), and its N- and C-terminal tails lie at opposite ends. They also found that ligand binding to Arc’s N-lobe did not cause major conformational changes to the rest of the protein [19]. Furthermore, Nielsen et al. produced the capsid domain of rat Arc (Arc^206-364^) that includes the N-lobe and the C-lobe, and solved its structure using the solution NMR method [20]. Recently, Cottee et al. and Erlendsson et al. studied the structure of Arc in fruit fly (*Drosophila*) using the x-ray crystallography and cryo-electron microscopy methods [21, 22]. Different from rat and other mammal species, the fruit fly has two copies of Arc protein: Arc1 and Arc2; Arc1 has 254 amino-acid residues and Arc2 has 193 amino-acid residues. Both Arc1 and Arc2 have a capsid domain, whose structure closely resembles the structure of the capsid domain of Arc in rat [21, 22]. In addition, Arc1 has a C-terminal tail, which is absent in Arc2. The tail contains a Cys-His zinc finger motif, and it can form an anti-parallel single-knuckle zinc finger with an adjacent Arc1 C-terminal tail [22]. However, the Arc protein in mammals does not have the zinc finger motif in its C-terminal tail.

In this study, we characterized the C-terminal tail of Arc in mammal species using the rat Arc protein as an example. We compared the sequences of Arc proteins in different mammals and found that the amino-acid composition in the C-terminal tail region has a significantly higher degree of variation rate than the rest of the protein. Structural prediction programs suggested that the C-terminal tail is structurally disordered. Chemical shift analysis based on solution NMR spectra confirmed that the C-terminal tail has a random coil (disordered) structure, and the tail starts from the residue D357. Furthermore, the NMR spectra showed that the C-terminal tail has minimum (if any) interaction with its neighboring capsid domain in Arc. Since the C-terminus of Arc in mammals has been relatively understudied, this study provides new information about a region of the protein that needs to be considered in the analysis of Arc structure-function relationships.

## Materials and methods

### Alignment of Arc protein sequences

The Arc protein sequences from different mammal species (human, chimpanzee, Rhesus monkey, rat, mouse, water buffalo, cattle, goat, and Arabian camel) were downloaded from the PubMed protein database (www.ncbi.nlm.nih.gov/pubmed) and compared with each other using the LALIGN algorithm [23]. In the alignment figure, the non-conserved amino-acid residues in the Arc protein sequence were highlighted in yellow color (the minority groups of residues were highlighted). The sequence segments and amino-acid residues that were found important for Arc functions were labeled above the protein sequence. The position of the secondary structures (mainly α-helices) in the Arc capsid domain (Arc^206-364^) were also labeled in the alignment figure.

### Prediction of disorder probability of Arc structure

Three algorithms, DISpro [24], IUPred [25], and PONDR-FIT [26], were used to predict the structurally ordered and disordered regions in Arc. The protein sequence of rat Arc (NCBI ID: NP_062234.1) was submitted to and analyzed by each of the three algorithms. As results, the disorder probability of each amino-acid residue in Arc was plotted against the amino-acid residue number of Arc. The sequence segments that have lower disorder probability values (< 0.5) are suggested to be structurally ordered (having structural domains), and the sequence segments that have higher disorder probability values (> 0.5) are suggested to be structurally disordered (having random coil structures).

### Production of Arc subdomain proteins

The DNA fragments encoding three rat Arc subdomain proteins (Arc^280-396^, Arc^208-396^, and Arc^208-363^) were respectively cloned into a pET32 vector using PCR methods. The pET32 vector adds a histidine-tag, a thioredoxin fusion protein, and a TEV cleavage sequence to the N-terminus of the produced Arc subdomain proteins. The DNA plasmids containing Arc subdomain sequences were transfected into BL21(DE3) cells, and the cells were grown in ^13^C-^15^N-labeled Spectra-9 media (Cambridge Isotope Laboratories, Inc.) at 37°C in a shaking incubator. When the optical density (OD_600_) of the cell culture reached 0.8, the temperature of the incubator was decreased to 20°C, and 1 mM of IPTG (isopropyl-β-d-thiogalactoside) solution was added to the cell culture to induce protein expression. The cells were harvested 17 hours after the addition of IPTG using centrifugation, and they were lysed on ice using sonication. The lysed cells were centrifuged at 10,000 rpm, and the supernatant after centrifugation was loaded to an AKTA Prime FPLC instrument (GE Healthcare, Inc.). The Arc subdomain proteins were purified from the supernatant using the nickel affinity, size-exclusion, and ion-exchange chromatography columns. Finally, the histidine-tag, thioredoxin fusion protein, and TEV (tobacco etch virus) protease cleavage sequence were removed from the Arc subdomain proteins using TEV protease.

### NMR

The NMR samples contained 500 μM of ^13^C-^15^N-labeled Arc subdomain proteins in a buffer with pH 7.0 containing 10 mM of Na_2_HPO_4_, 50 mM of NaCl, 2mM of TCEP, 0.05% of NaN_3_, and 10% of D_2_O. NMR experiments were performed on a Bruker 600 or 800 MHz spectrometer equipped with TCI triple-resonance cryogenic probes using standard Bruker pulse programs. ^1^H, ^13^C, and ^15^N backbone resonances were assigned using standard triple resonance experiments, such as TROSY, HNCA, HNCACB, CBCA(CO)NH, and HN(CO)CA. All of the 3D experiments were collected with 16 transients at 293 K. All of the spectra were processed using the Topspin software (Bruker, Inc.) and analyzed using the Computer-Aided Resonance Assignment (CARA) software [27].

### Chemical shift calculation

The chemical shift of the α-carbon of each amino-acid residue in Arc^280-396^ was extracted from its NMR-HNCA spectra. The chemical shift was compared to the standard chemical shift of the α-carbon of the same amino-acid residue in a random coil structure. The difference in chemical shift (ΔC_α_, ppm) of each amino-acid residue was calculated and plotted against the amino-acid residue number of Arc^280-396^. The continuous and significantly positive ΔC_α_ values (> 0.05) suggest that the corresponding sequence segments have an α-helix secondary structure. The continuous and significantly negative ΔC_α_ values (< -0.05) suggest that the corresponding sequence segments have a β-strand secondary structure. The discrete and insignificant ΔC_α_ values (between -0.05 to 0.05) suggest that the corresponding sequence segments have a random coil structure.

## Results

### The amino-acid residue composition of Arc C-terminal tail is highly divergent

**Fig 1:**
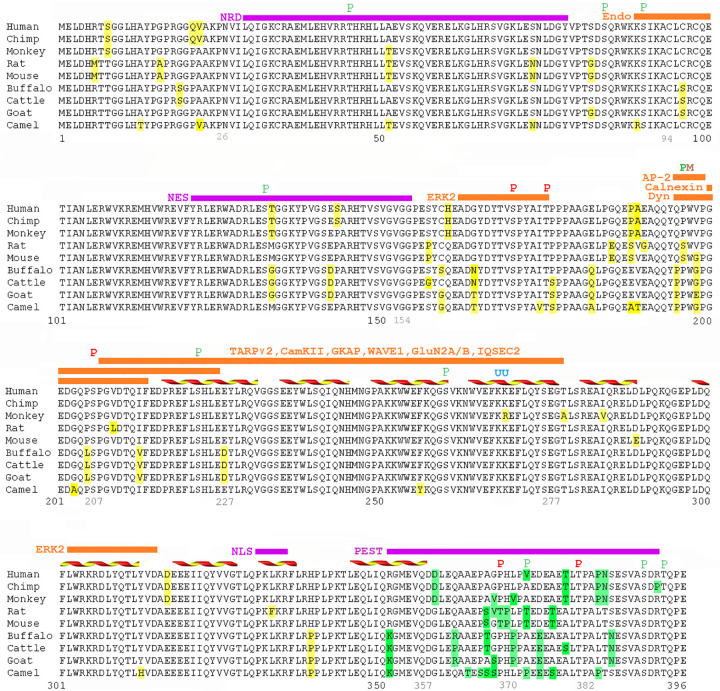
Sequence alignment of Arc proteins in different mammal species. The sequence of Arc proteins in different mammal species were aligned and compared with each other. The non-conserved amino-acid residues in the protein sequences were highlighted in green color (in the C-terminal sequence segment Arc^351-396^) or yellow color (in the rest of the protein). The alignment result shows that the amino-acid residue composition in the C-terminal tail region of the Arc protein (~ Arc^351-396^) is highly divergent compared to the rest of the protein. The purple bars above the protein sequence indicate the sequence regions that are important for Arc’s nuclear transportation (NRD, NES, NLS) [14] and degradation (PEST) [28]; the orange bars indicate the sequence regions that are important for Arc’s interaction with other proteins (these proteins’ names were labeled before/above the orange bars) [10, 11, 13, 15, 20, 29–33]; the colored letters indicate Arc’s phosphorylation sites (red “P”) [31, 34], putative phosphorylation sites (green “P”) [1, 2], ubiquitination sites (blue “U”) [13], and mutation site (brown “M”) [30]; the yellow-red ribbons indicate the α-helix secondary structures in Arc’s capsid domain [15, 20]. (See the Discussion section for more detail).

In this study, we aligned and compared the sequences of Arc proteins in different mammal species. The alignment result revealed the conserved and non-conserved amino-acid residues (Fig 1). It is noticeable that the amino-acid composition in the C-terminal tail region has a significantly higher degree of variation rate than the rest of the protein. Specifically, the variation rate is 39.1% in the C-terminal sequence segment Arc^351-396^ (the residue variations highlighted in green color); whereas, the average variation rate is 12.3% in the rest of the protein (the residue variations highlighted in yellow color). It is commonly believed that the more functionally important regions in a protein are more structurally ordered (having structural domains), and their sequence compositions are more conserved during biological evolution; on the other hand, the less functionally important regions have more disordered structures (having random coil structures) and their sequence compositions are less conserved during evolution. The high degree of variation rate of amino-acid composition in Arc^351-396^ could suggest that this region is less functionally important and is more structurally disordered. As a comparison to the C-terminal tail region, the variation rate of amino-acid composition of Arc’s N-lobe (Arc^207-277^) is only 7.0%. The N-lobe is involved in Arc’s interaction with several other proteins, and it has a highly ordered structure. Furthermore, the alignment result revealed some features of Arc protein, such as the phosphorylation sites S84 and S170, that are common among mammal species. This information adds to our understanding of how the sequences of Arc proteins from different animal classes compare with one other [18].

### The C-terminal tail of Arc is predicted to be structurally disordered

**Fig 2:**
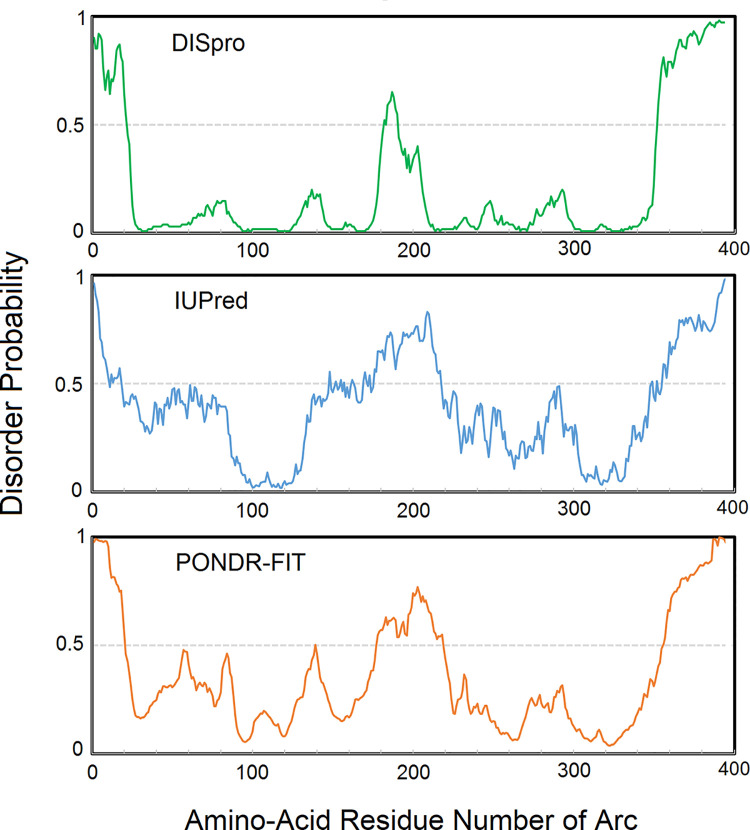
Prediction of structural disorder probability of Arc protein. The disorder probability of each amino-acid residue in Arc was plotted against the amino-acid residue number of Arc. The C-terminal tail of Arc was consistently predicted to have larger disorder probability values (> 0.5) by three prediction algorithms. The larger disorder probability suggests that the corresponding sequence segment is structurally disordered (having random coil structure).

Three prediction algorithms (DISpro, IUPred, and PONDR-FIT) were used to analyze the protein sequence of Arc (Fig 2). These algorithms predict each sequence segment’s probability of being structurally ordered or disordered. A disorder probability value higher than 0.5 suggests that the corresponding sequence segment of a protein tends to be structurally disordered; whereas, a disorder probability value lower than 0.5 suggests that the corresponding sequence segment tends to be structurally ordered. All three prediction algorithms consistently predicted that the C-terminal tail region of the Arc protein (approximately Arc^356-396^) has high disorder probability values (> 0.5), suggesting this region is likely to be structurally disordered. As a comparison to the C-terminal tail region, the prediction algorithms consistently predicted that the sequence segment Arc^210-350^ has low disorder probability values (< 0.5), suggesting this region is structurally ordered. Indeed, the sequence segment Arc^210-350^ largely overlaps with the structurally ordered capsid domain (Arc^206-364^). In addition, the prediction algorithms consistently predicted that the N-terminal tail and the middle region of the Arc protein have high disorder probability values (> 0.5), suggesting they could also be structurally disordered. The prediction algorithms also consistently predicted that the region centered around the sequence segment Arc^90-120^ has low disorder probability values (< 0.5), suggesting this region could be structurally ordered. However, the exact sequence ranges of the N-terminal tail, the middle region, and the potentially structured region between them were not consistently defined. The prediction result adds to knowledge and complement with the previous study [18].

### The C-terminal tail of Arc has a random coil structure and the tail starts at residue D357

**Fig 3:**
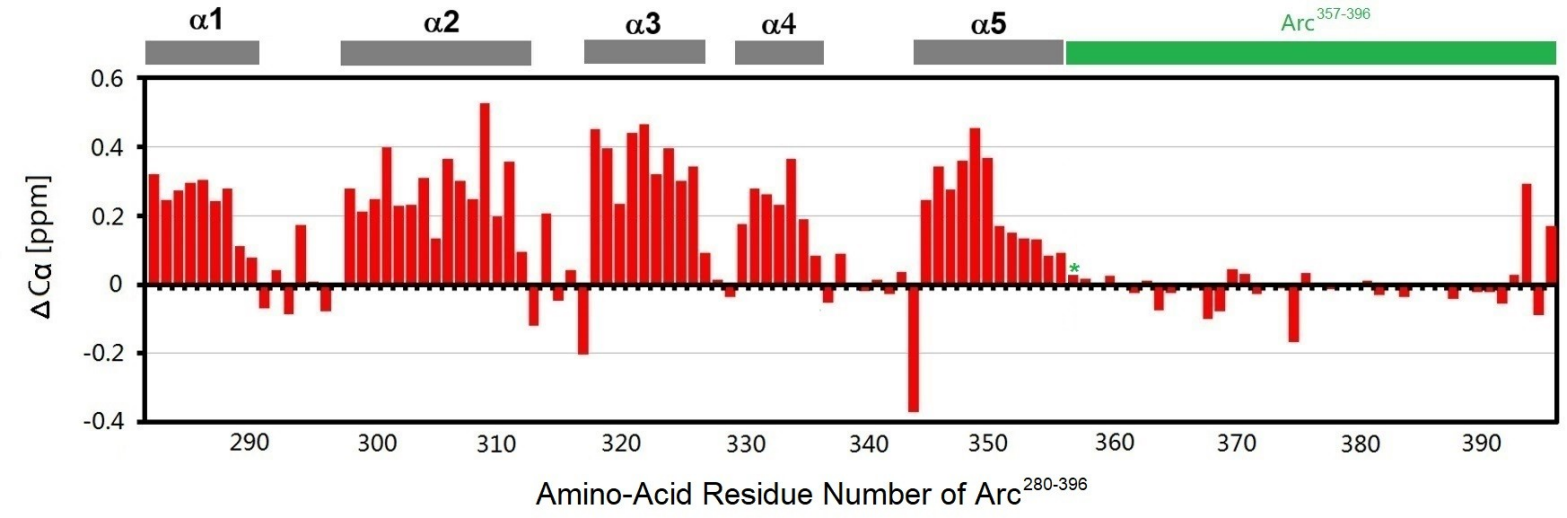
NMR chemical shift analysis of Arc^280-396^. The chemical shift differences of α-carbon (ΔC_α_) were plotted against the amino-acid residue number of Arc^280-396^. The sequence segments with continuous and significantly positive ΔC_α_ values (> 0.05) correspond to the five α-helices (grey bars, α1-α5) in the C-lobe region. The C-terminal tail (green bar) has insignificant (-0.05 ~ 0.05) or discrete ΔC_α_ values, suggesting this region has a random coil structure. The chemical shift data of Arc^280-396^ was deposited in the Biological Magnetic Resonance Data Bank (BMRB) (ID: 50436).

In order to experimentally determine the structure of Arc C-terminal tail, we produced the ^13^C-^15^N-labeled Arc^280-396^ protein, which includes the C-lobe subdomain and the previously uncharacterized C-terminal tail. We collected a series of 3D NMR spectra on the protein, and the α-carbon chemical shift of each amino-acid residue in Arc^280-396^ was determined from the NMR spectra (except the first two amino-acid residues and the proline residues). The chemical shift was compared to the standard chemical shift of the α-carbon of the same amino-acid residue in a random coil structure. The difference in chemical shift (ΔC_α_, ppm) of each amino-acid residue was calculated and plotted against the protein sequence of Arc^280-396^ (Fig 3). The continuous and significantly positive ΔC_α_ values (> 0.05) suggest that the corresponding sequence segments have an α-helix secondary structure, such as the five α-helices in Arc^280-396^ (grey bars, α1-α5) that agrees with the five-helix bundle structure of Arc C-lobe subdomain [15]. The discrete and insignificant ΔC_α_ values (between -0.05 to 0.05) suggest that the corresponding sequence segments have a random coil (disordered) structure, such as the Arc C-terminal tail Arc^357-396^ (green bar). Furthermore, the plot does not contain continuous and significantly negative ΔC_α_ values (< -0.05), which suggests that the Arc^280-396^ protein does not have β-strand secondary structure.

### The C-terminal tail has minimal interaction with its neighboring capsid domain

**Fig 4:**
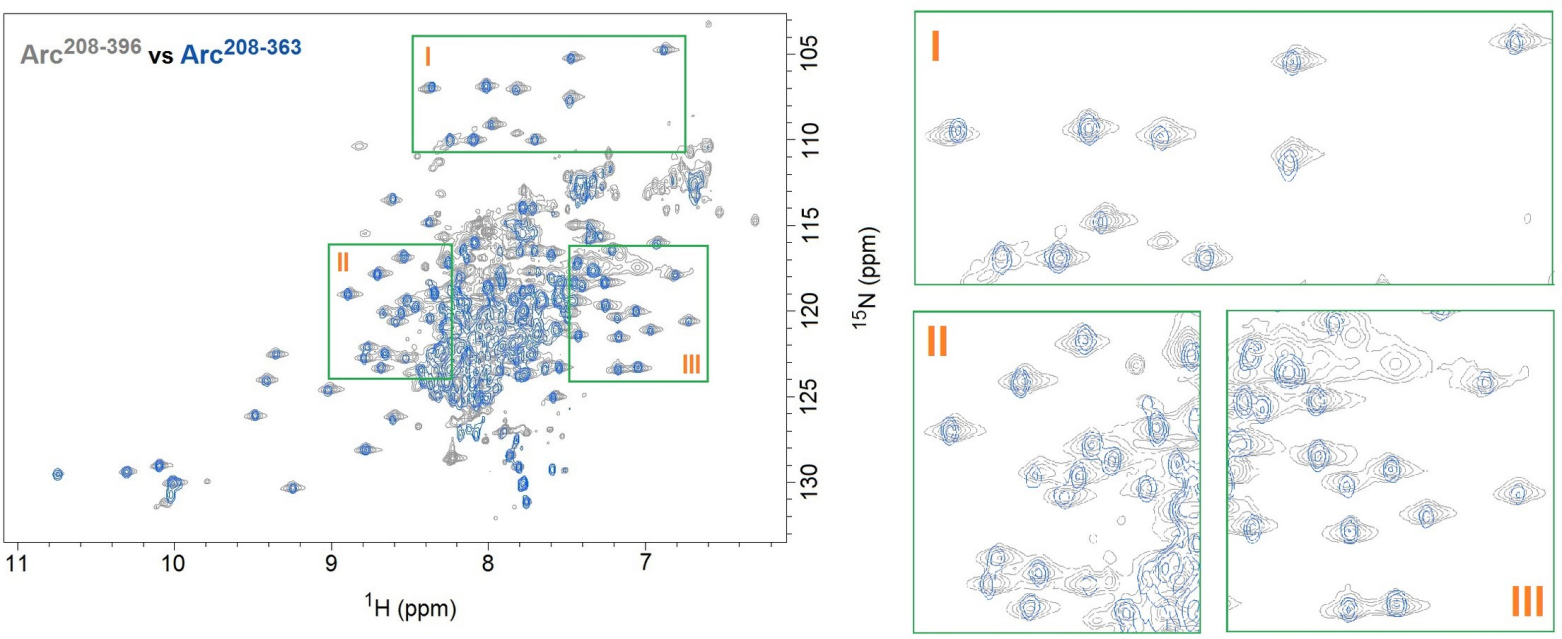
Comparison of NMR TROSY spectra of Arc^208-396^ and Arc^208-363^. The Arc^208-396^ protein (grey) has the additional C-terminal tail compared to the Arc^208-363^ protein (the capsid domain, blue). The presence of the C-terminal tail does not generate noticeable chemical shifts on the capsid domain peaks, suggesting the C-terminal tail has minimum (if any) interaction with the capsid domain. (The green boxes I, II, and III on the right are enlarged views of the example regions in the TROSY spectra on the left).

**Fig 5:**
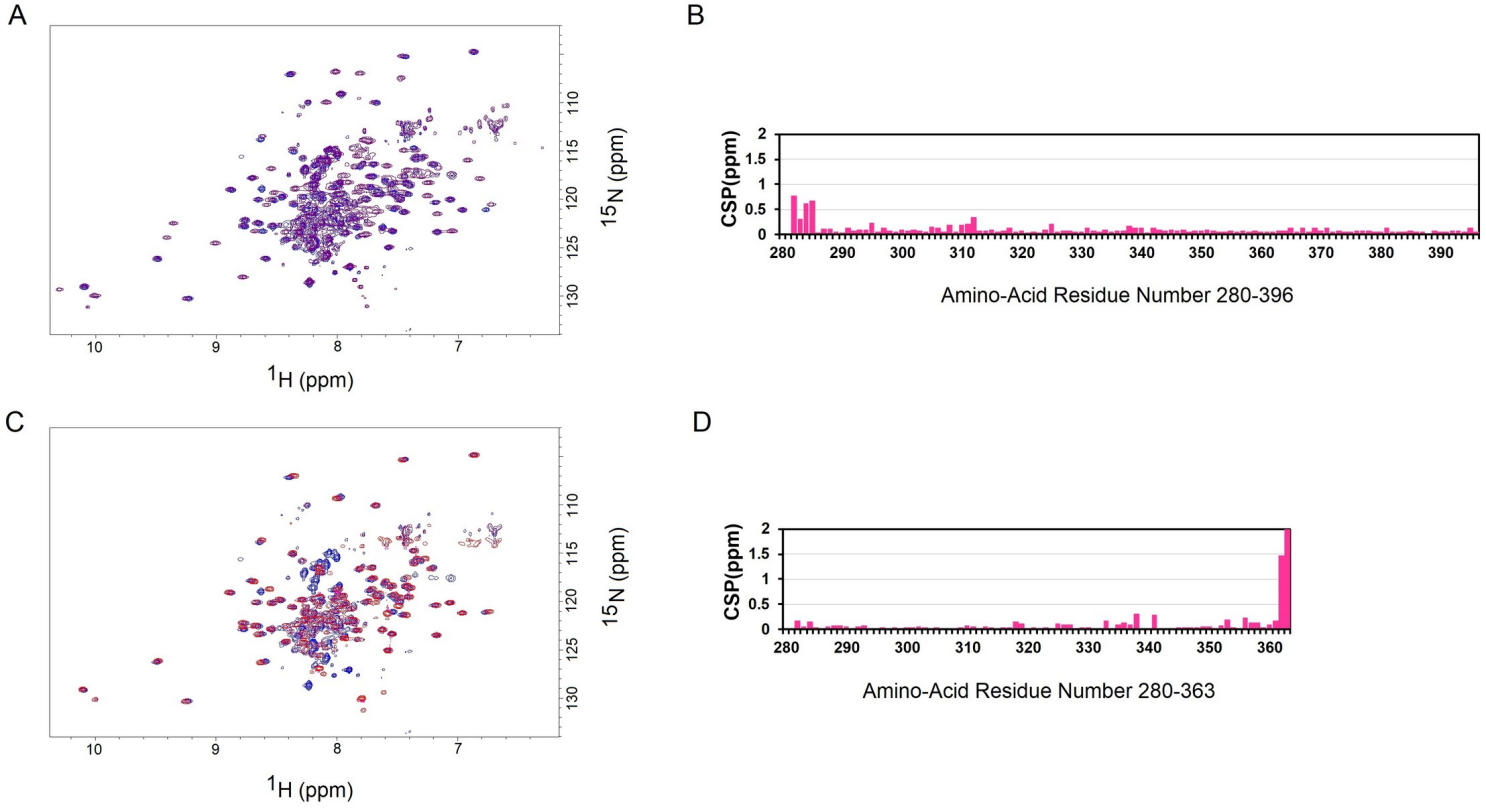
Changes of the chemical shifts of the backbone amide resonance between Arc^280-396^ and Arc^208-396^/Arc^280-363^. (A) The overlay of NMR TROSY spectra of Arc^280-396^ (blue) and Arc^208-396^ (purple). (B) The chemical shift perturbations (CSP) of residues 280–396 when the amide chemical shifts of Arc^208-396^ and Arc^280-396^ are compared with each other. (C) The overlay of NMR TROSY spectra of Arc^280-396^ (blue) and Arc^280-363^ (red). (D) The chemical shift perturbations (CSP) of residues 280–363 when the amide chemical shifts of Arc^280-363^ and Arc^280-396^ are compared with each other. The CSPs of backbone amides are calculated using the equation [(ΔH)^2^+(ΔN)^2^/5]^1/2^, where ΔH and ΔN are the changes in the ^1^H and ^15^N chemical shifts respectively. The chemical shift data were deposited in the BMRB database (BMRB ID: 50436 (Arc^280-396^), 50440 (Arc^208-396^), and 50441 (Arc^280-363^)).

We tested if there is any interaction between the C-terminal tail and the capsid domain adjacent to it. We successfully produced the capsid domain protein (Arc^208-363^), but our attempts to produce the C-terminal tail (Arc^357-396^) failed as this disordered protein was degraded during bacterial expression. As an alternative approach, we produced the Arc^208-396^ protein, which includes the capsid domain and the C-terminal tail. We collected the NMR TROSY spectra of Arc^208-363^ and Arc^208-396^, and compared them with each other (Fig 4). The TROSY spectrum of Arc^208-363^ (blue) almost precisely overlaps with the TROSY spectrum of Arc^208-396^ (grey) (except the latter has additional cross-peaks from its C-terminal tail). In addition, we compared the NMR TROSY spectra of Arc^280-396^ and Arc^208-396^ (Fig 5A), and analyzed their chemical shifts of the backbone amide resonance (Fig 5B). Arc^280-396^ includes the C-lobe and C-terminal tail; Arc^208-396^ has an additional N-lobe than Arc^280-396^. The analysis result showed that the presence/removal of residues 208–279 (N-lobe) did not cause major chemical shift perturbation (CSP) in residues 280–396 (C-lobe and C-terminal), except for residues 282–285 that connect N-lobe and C-lobe (Fig 5B). It suggests that the N-lobe has minimum (if any) interaction with the C-terminal tail (and the C-lobe). We also compared the chemical shifts of the backbone amide resonances between Arc^280-396^ and Arc^280-363^ (Arc^280-396^ includes the C-lobe and C-terminal tail; Arc^280-363^ includes only the C-lobe) (Fig 5C). The comparison result showed that the presence/removal of residues 364–396 (C-terminal tail) did not cause major chemical shift perturbation (CSP) in residues 280–363 (C-lobe), except for residues 362–363 that connect C-lobe and C-terminal tail (Fig 5D). It suggests that the C-lobe also has minimum (if any) interaction with the C-terminal tail. Taken together, these data suggest that the C-terminal tail has minimum (if any) interaction with the N-lobe and C-lobe (together they form the capsid domain), and it does not affect the folding of the capsid domain. This observation agrees with a previous finding that the C-terminal tail lies outside the core of the Arc protein, which is consisted of the Arc N-terminus sitting above the capsid domain [19]. However, further experiments are required to examine the possible interaction between the C-terminal tail and the capsid domain.

## Discussion

Arc was discovered in 1995, and it was found to play an important role in synaptic plasticity and memory formation. Solving the structure of Arc will help understand its functional mechanism. In this study, we characterized the C-terminal tail of Arc in mammals using the rat Arc protein as an example. We found that the C-terminal tail of Arc in mammal species has a significantly higher variation rate in amino-acid composition than the rest of the protein (39.1% vs. 12.3%), suggesting this region may lack important functional domains and/or ordered structures. The C-terminal tail was predicted to have high disorder probability, suggesting this region is structurally disordered. The NMR chemical shift analysis of Arc^280-396^ showed that the C-terminal tail indeed has a random coil (disordered) structure, and the tail starts from the residue D357. We further tested if the tail interacts with the neighboring capsid domain and found that it has minimum (if any) interaction with the latter.

In the recently characterized structure of *Drosophila* Arc1, 240 copies of Arc1 oligomerize into 12 pentameric and 30 hexameric capsomeres, and these capsomeres further form an icosahedral capsid sphere. Arc1’s 48-residue C-terminal tail locates inside the capsid and below the capsomeres. The residues 224–252 of two C-terminal tails under each hexameric capsomere form anti-parallel zinc fingers; whereas, the other residues in the two C-terminal tails and the rest copies of C-terminal tail are structurally disordered [22]. The C-terminal tail of Arc1 also contains 12 basic residues; together with the zinc fingers, they may facilitate Arc1’s mRNA recognition and binding, similar to the function of the nucleocapsid domain of the retrovirus HIV [22]. For the C-terminal tail of Arc in mammals, it may also locate inside the capsid as the *Drosophila* Arc1, and it could interfere with the capsid formation of the full-length Arc as the capsid domain alone was found unable to form the capsid layer [17]. But it lacks the zinc fingers and basic residue patches for mRNA regulation, and no close homology to its sequence was found in other retrotransposons or retroviruses either.

**Fig 6:**
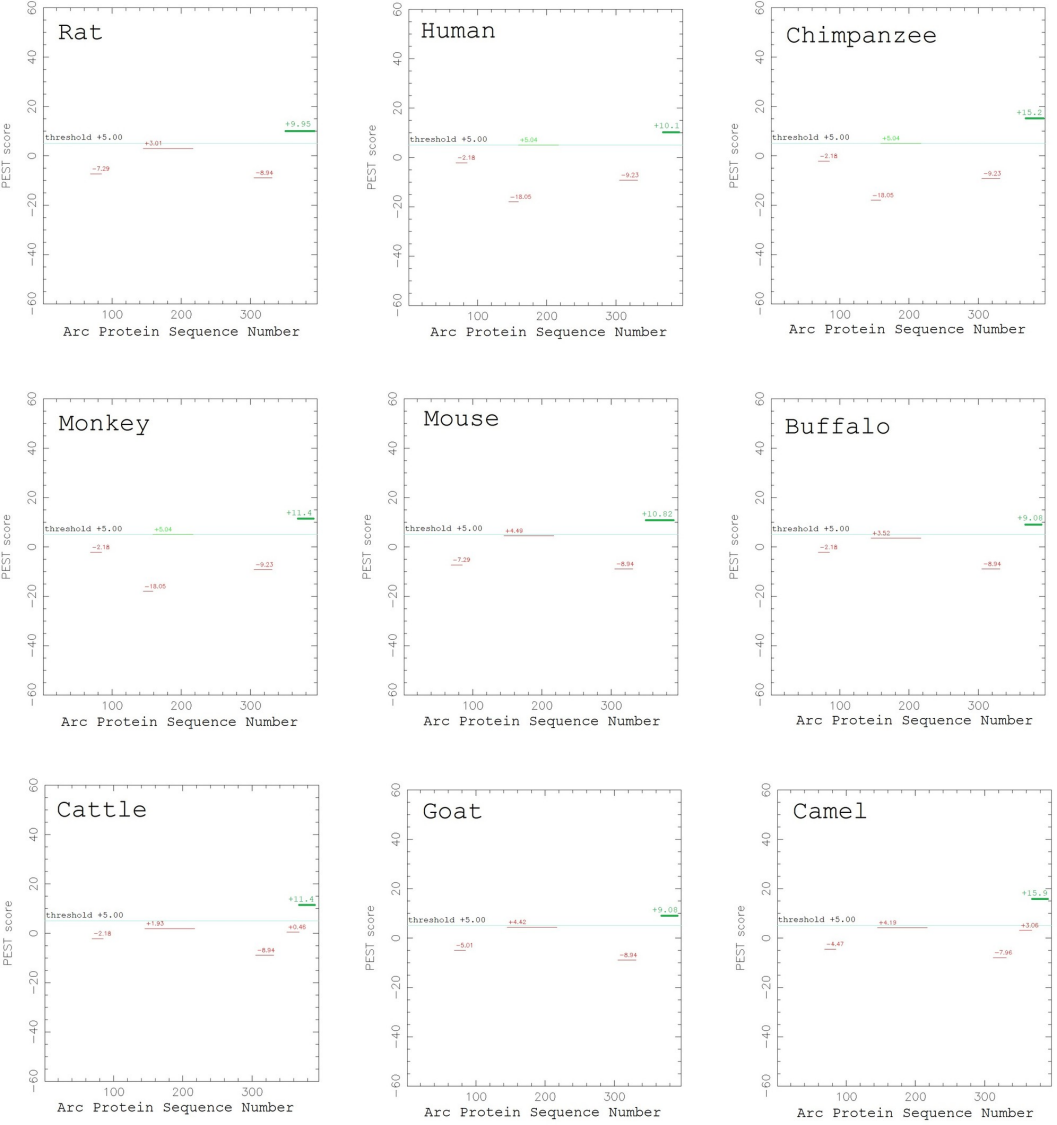
The PEST signal region in Arc C-terminal tail. The C-terminal tail of rat Arc contains a PEST signal region (the green bar that reads “+9.95” in the upper left plot). This region is largely preserved in the other examined mammal species (the green bars in the other 8 plots). The PEST score plots were generated using the ePESTfind tool at https://emboss.bioinformatics.nl/cgi-bin/emboss/epestfind.

To identify the possible functions of the C-terminal tail of Arc in mammals, we reviewed the previous studies that examined the functions of different sequence segments of Arc. These studies have shown that, within the C-terminal tail (Arc^357-396^), residues 351–392 of Arc were predicted as a PEST signal region (a sequence segment that is rich in P, E, S, T residues) using the ePESTfind program [35], which may contribute to the protein’s proteasome-dependent degradation [28]. Although the original PEST signal analysis was conducted on the Arc protein in rat, our sequence alignment result in this study showed that the PEST signal has been largely preserved in other mammal species despite the high variation rate of amino-acid composition at the C-terminal tail (Fig 6). In addition, phosphorylation sites have been identified in the C-terminal tail at residues T368, and T380 [31, 34]. It was also predicted that this region contains putative phosphorylation sites by the protein kinase C at residues S390, and by the casein kinase II at residues T372 and T393 [1, 2]. Among these phosphorylation sites, our sequence alignment result showed that the sites T380, S390, and T393 are preserved in the mammal species; whereas the sites T368 and T372 are only present in rat and mouse.

Furthermore, previous studies have shown that, for the Arc sequence segments that partially overlap with or include the C-terminal tail (Arc^357-396^), residues 228–380 of Arc share 20% sequence similarity with the 21st and 22nd repeats of the α-spectrin protein [1, 2]; since these structural motifs of α-spectrin tend to form oligomers [36], the corresponding 228–380 region of Arc may contribute to its self-oligomerization [18], and the Arc oligomers may further contribute to memory formation [37]. In addition, residues 155–396 of Arc bind to the dynamin 2 protein [10] and the clathrin-adaptor protein 2 (AP-2) [30] to regulate AMPA receptor endocytosis; residues 94–382 of Arc bind with the Triad3A protein, and Triad3A can ubiquitinate Arc at its residues K268 and K269 to help regulate synaptic strength [13]. Arc was also found to interact with Tip60 [38], PICK1 (especially its BAR domain, residues 152–278) [39], GSK3α/β [34], and CaM kinase II [3, 12]; however, the specific sequence segments of Arc that regulate these interactions have not been identified. Therefore, the C-terminal tail of Arc may also contribute to Arc’s self-oligomerization process as part of the spectrin-homologous domain, or in Arc’s interaction with other proteins, such as dynamin 2, AP-2, Triad3A, Tip60, PICK1, GSK3α/β, and/or CaM kinase II. Taken together, we speculate that, although the C-terminus is disordered, it may play important functional roles that vary between species.
